# The Prevalence of Antibiotic Resistance Phenotypes and Genotypes in Multidrug-Resistant Bacterial Isolates from the Academic Hospital of Jaén, Spain

**DOI:** 10.3390/antibiotics13050429

**Published:** 2024-05-09

**Authors:** Laura Morales, Antonio Cobo, María Pilar Frías, Antonio Gálvez, Elena Ortega

**Affiliations:** 1 Microbiolgy Unit, Department of Health Sciences, Faculty of Experimental Sciences, University of Jaén, 23071 Jaén, Spain; lmh00003@red.ujaen.es (L.M.); acmolinos@ugr.es (A.C.); agalvez@ujaen.es (A.G.); 2 Department of Statistics and Operation Research, Faculty of Experimental Sciences, University of Jaén, 23071 Jaén, Spain; mpfrias@ujaen.es

**Keywords:** multidrug-resistant bacteria, genetic determinants of resistance, clinical isolates

## Abstract

The heterogenicity of antimicrobial resistance genes described in clinically significant bacterial isolates and their potential role in reducing the efficacy of classically effective antibiotics pose a major challenge for global healthcare, especially in infections caused by Gram-negative bacteria. We analyzed 112 multidrug-resistant (MDR) isolates from clinical samples in order to detect high resistance profiles, both phenotypically and genotypically, among four Gram-negative genera (*Acinetobacter*, *Escherichia*, *Klebsiella,* and *Pseudomonas*). We found that 9.8% of the total selected isolates were classified as extensively drug-resistant (XDR) (six isolates identified as *A. baumannii* and five among *P. pneumoniae* isolates). All other isolates were classified as MDR. Almost 100% of the isolates showed positive results for *bla*_OXA-23_ and *bla*_NDM-1_ genes among the *A. baumannii* samples, one resistance gene (*bla*_CTX-M_) among *E. coli*, and two genetic determinants (*bla*_CTX-M_ and *aac(6′)-Ib*) among *Klebsiella*. In contrast, *P. aeruginosa* showed just one high-frequency antibiotic resistance gene (*dfrA*), which was present in 68.42% of the isolates studied. We also describe positive associations between ampicillin and cefotaxime resistance in *A. baumannii* and the presence of *bla_VEB_* and *bla_GES_* genes, as well as between the aztreonam resistance phenotype and the presence of *bla_GES_* gene in *E. coli*. These data may be useful in achieving a better control of infection strategies and antibiotic management in clinical scenarios where these multidrug-resistant Gram-negative pathogens cause higher morbidity and mortality.

## 1. Introduction

The discovery of antimicrobial compounds, as well as their routine administration in clinic to treat bacterial infections, revolutionized modern medicine during the 20th century, changing the therapeutic paradigm. In fact, antibiotics have been one of the most important medical advances for the development of medical areas such as organ transplantation, basic and complex surgical procedures, management of patients with infectious diseases, or cancer treatment [1]. Antibiotics have played a very important role fighting against a myriad of infectious microbes for decades, and it is high time to target the principal causes of the global antibiotic resistance problem, as antibiotic-resistant bacteria today have the potential of taking the lives of above 700,000 individuals per year [2,3].

The emergence of resistance and especially multidrug resistance among the most important bacterial pathogens is recognized as a major public health threat affecting humans worldwide and one of the major global healthcare crises in our current era [4]. The overuse and misuse of antibiotics, together with the lack of new drugs in development, as well as the absence of diagnostic testing before prescribing antibiotics, are the main factors of this issue, so nowadays, antibiotic-resistant strains strongly hinder clinical treatments [5,6]. In fact, the World Health Organization (WHO) estimates that in 2050, antibiotics will not be available, and the total deaths due to multidrug-resistant bacteria will be around 10 million, which is more than the total deaths from cancer disease per year [5,7,8].

The present goal of many researchers is to develop solutions to this problem of multidrug resistance, which remains a global priority. Different mechanisms of bacterial resistance have been reported recently for almost all the antibiotics used in human clinics [9]. The spread of resistance is mainly caused by the rapid replication of bacterial cells and the process of conjugation, which helps in the transfer of antibiotic-resistant plasmid genes among bacteria [10,11].

The different types of antimicrobial resistance genes and their possible variants differ geographically. Therefore, it is very important to investigate the predominant resistance mechanisms around us in order to choose the appropriate antimicrobials in future treatments [12]. Over the last few years, a multitude of new genes related to antibiotic resistance have been described, such as *bla*_NDM-1_, *bla*_OXA-23_, *bla*_OXA-64_, *bla*_PER-7_, and *bla*_ADC-57_ (related to carbapenemases and Metallo-Beta-lactamases) or *tet* (X3) and *tet* (X4) genes (related to tetracycline and tigecycline resistance) [13,14,15,16,17]. Resistance to colistin through the plasmid-mediated *MCR-1* gene is the most recently described, raising concerns about the development of Gram-negative bacteria that are completely resistant to antibiotics [18].

An example of this rapid emergence and dissemination of antibiotic resistance is carbapenem resistance in Gram-negative bacilli, which has posed real problems in the treatment of infections caused by these bacteria. The World Health Organization recognizes *Enterobacterales* that are resistant to carbapenems or producers of extended spectrum beta-lactamases (ESBL), as well as non-fermenters (*Pseudomonas aeruginosa* and *Acinetobacter baumannii*) that are resistant to carbapenems [19,20,21,22], as critical priority bacteria, for which we urgently need new antimicrobials. Moreover, carbapenem-resistant *A. baumannii* (CRAB) have been classified by the World Health Organization (WHO) as one of the “Priority 1: Critical group” organisms for which new antimicrobials are urgently needed. In this way, the emergence of *A. baumannii* that are resistant to carbapenem has been very significant, not only because of the resistance acquired but also because of the dissemination of the bacteria in the population [23].

The main mechanisms of carbapenem resistance in *A. baumannii* are OXA-type carbapenemases, but it is also important to analyze the combination of resistance genes and the availability of alternative therapeutic options, which represent an important challenge in deciding on their appropriate management. In this case, not only the molecular determinants of carbapenem resistance must be taken into consideration, but also the combination of resistance to different antibiotics, which may also increase global resistance levels in pathogens [17,24]. The same concern may be applied to other Gram-negative bacteria, such as *Escherichia* or *Klebsiella* species. The development and spread of resistance to ß-lactams, together with the broad antimicrobial spectrum of fluoroquinolones, led to the use of the latter drugs as an empirical therapy for a wide variety of both community-acquired and nosocomial infections. Particularly, the massive use of quinolones has caused increased levels of resistance to fluoroquinolones, so that in Spain, the rate of *Escherichia coli* isolates that are resistant to fluoroquinolones has increased by more than 16% in recent years [25]. The Spanish Society of Infectious Diseases and Clinical Microbiology (SEIMC) published in 2015 a Clinical Guideline on the management of invasive infections due to multidrug-resistant *Enterobacteriaceae*, including carbapenem-resistant *Enterobacterales* (CRE) or carbapenemase-producing *Enterobacterales* (CPE), carbapenem-resistant *Pseudomonas aeruginosa* (CR-PA), and carbapenem-resistant *Acinetobacter baumannii* (CR-AB) [26], but there are still few studies about the phenotypic or genotypic antibiotic resistance prevalence in clinical samples in Spain.

We analyzed 112 multidrug-resistant isolates from clinical samples in order to detect high resistance profiles, both phenotypically and genotypically, among four Gram-negative genera (*Acinetobacter*, *Escherichia*, *Klebsiella*, and *Pseudomonas*). We also searched for possible positive associations between the presence of antibiotic resistance genes and the XDR/MDR phenotype [27].

## 2. Results

### 2.1. Bacterial Isolates

Samples obtained at the Microbiology Unit of the Academic Hospital in Jaén (Spain) and subjected to antimicrobial resistance tests were reviewed over 9 months throughout the year 2019, and multidrug-resistant isolates were selected to be included in this study.

Of these samples, 65.2% were from male patients and 34.8% from female patients. The patients were divided into four age groups: there were no samples from children (0–14 years old), 3.6% of samples were from youths (15–40 years old), 29.5% were from middle-aged patients (41–60 years old), and 66.9% were from older patients (>60 years old).

With regard to the origin of the isolates, they were mainly from 28 different types of clinical samples. Respiratory samples (bronchial aspirate, tracheal aspirate, sputum, and respiratory samples) were prevalent in the isolation of multidrug-resistant isolates of Acinetobacter (38.8% of the total clinical samples analyzed) and *Pseudomonas* (52.6%), and 19.3% of the samples were related to urine (urine, mid-micturition urine, bladder puncture urine, bladder catheter urine, and ureter urine), where multidrug-resistant *Acinetobacter* were also isolated. Multidrug-resistant isolates of the genera *Escherichia* and *Klebsiella* were mainly isolated from clinical samples of a urine origin, with 72% and 39%, respectively. In addition, a high percentage of multidrug-resistant isolates of *Klebsiella* (26.2%) were also isolated from samples of a blood origin (blood, arterial blood, central venous catheter blood, venipuncture blood, and blood culture) (Table 1).

Among the 197 total samples, we selected 112 bacterial isolates identified as MDR *A. baumannii*, *E. coli*, *K. pneumoniae,* and *P. aeruginosa* based on antimicrobial resistance tests and MALDI-TOF identification.

Among them, 31 were confirmed as *A. baumannii*, 25 as *E. coli*, 37 as *K. pneumoniae,* and 19 as *P. aeruginosa* by 16S ribosomal DNA V3–V5 Sequence Identification (Table 2, Table 3, Table 4 and Table 5).

### 2.2. Antimicrobial Resistance

**Figure 1:**
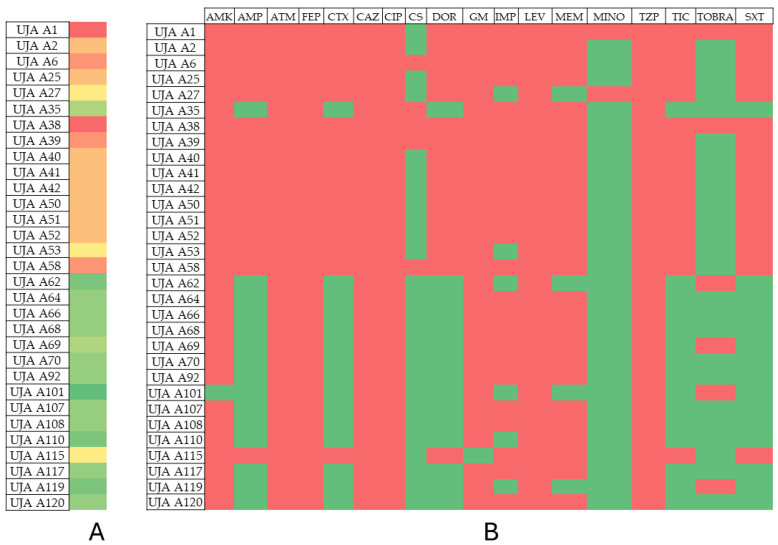
Prevalence of antibiotic resistance in *Acinetobacter baumannii*. (**A**) Profile of resistance of each isolate. Color scales from red to green indicate rank from higher to lower number of antibiotic resistance phenotype; (**B**) prevalence of antibiotic resistance among isolates. Red: resistant; green: susceptible.

**Figure 2:**
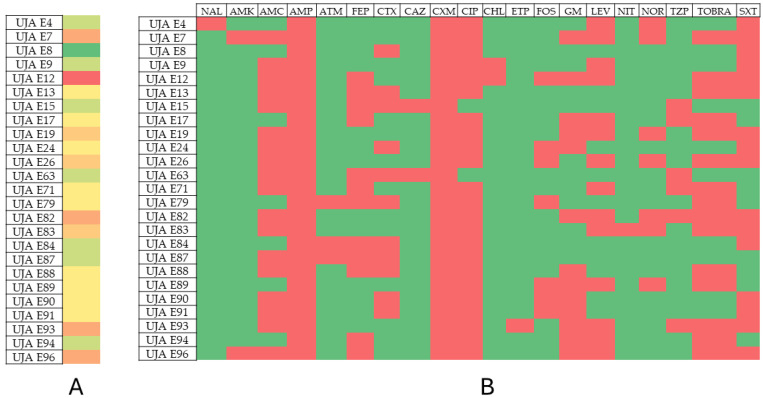
Prevalence of antibiotic resistance in *Escherichia coli*. (**A**) Profile of resistance of each isolate. Color scales from red to green indicate rank from higher to lower number of antibiotic resistance phenotype; (**B**) prevalence of antibiotic resistance among isolates. Red: resistant; green: susceptible.

**Figure 3:**
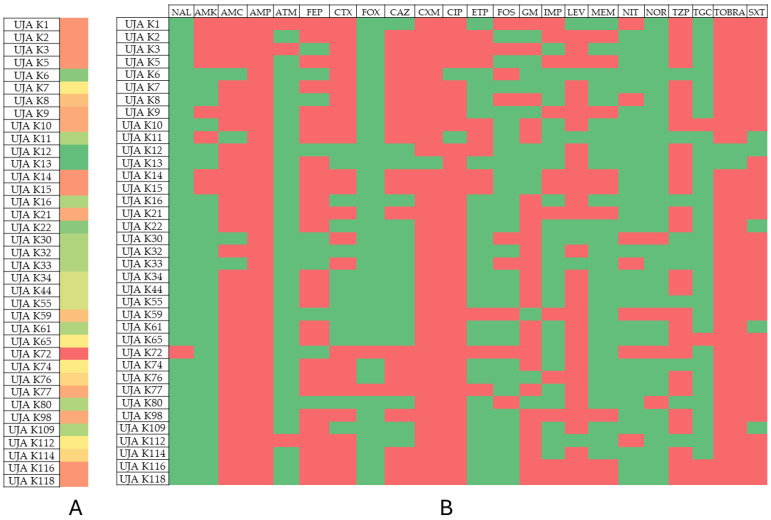
Prevalence of antibiotic resistance in *Klebsiella pneumoniae*. (**A**) Profile of resistance of each isolate. Color scales from red to green indicate rank from higher to lower number of antibiotic resistance phenotype; (**B**) prevalence of antibiotic resistance among isolates. Red: resistant; green: susceptible.

**Figure 4:**
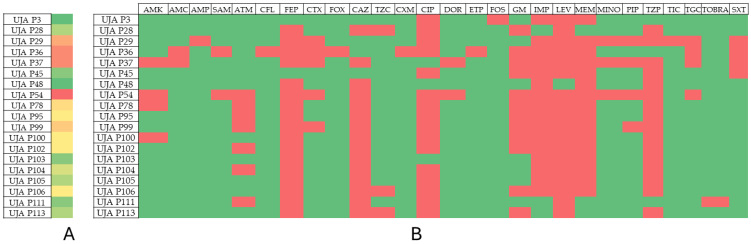
Prevalence of antibiotic resistance in *Pseudomonas aeruginosa*. (**A**) Profile of resistance of each isolate. Color scales from red to green indicate rank from higher to lower number of antibiotic resistance phenotype; (**B**) prevalence of antibiotic resistance among isolates. Red: resistant; green: susceptible.

The results of the phenotypic and genotypic resistance profiles of the analyzed isolates are shown in Table 2, Table 3, Table 4 and Table 5. Figure 1, Figure 2, Figure 3 and Figure 4 show the prevalence of antibiotic resistance among the four genera studied.

Among the 112 selected isolates, 6 isolates were identified as *A. baumannii* (UJA A1, UJA A6, UJA A27, UJA A38, UJA A39, and UJA A58), and 5 were identified as *P. aeruginosa* (UJA P54, UJA P78, UJA P95, UJA P99, and UJA P102), which includes 9.8% of the total selected isolates being classified as extensively drug-resistant (XDR), meaning that these isolates were non-susceptible to at least one agent in all but two antimicrobial categories, as described by Magiorakos et al., 2012 [27]. On the other hand, 25 isolates of *Acinetobacter*, 14 belonging to *Pseudomonas* genera, as well as all the isolates identified as *K. pneumoniae* and *E. coli,* were classified as multidrug-resistant, as the isolates were non-susceptible to at least one agent in ≥3 antimicrobial categories [27].

Concerning specific isolates, UJA A1 and UJA A38 stand out for being resistant to 17 antibiotics, and isolates UJA A6, UJA A39, and UJA A58 stand out for being resistant to 16 of the tested antibiotics. Eight isolates also showed resistance to 15 antibiotics (UJA A2, UJA A25, UJA A40, UJA A41, UJA A42, UJA A50, UJA A51, and UJA A52). In contrast, isolate UJA A101 showed resistance to eight antibiotics, being the least resistant among this group.

Among the *Pseudomonas* isolates, it is worth highlighting isolate UJA P54, which showed resistance to 17 antibiotics, as well as isolates UJA P36 and UJA P37, with resistance to 15 antibiotics. However, resistance to only five antibiotics was found in isolates UJA P3 and UJA P48 and to six antibiotics in isolates UJA P45, UJA P103, and UJA P111. 

On the other hand, *Escherichia* and *Klebsiella* isolates showed 100% resistance to Ampicillin and more than 90% resistance to other antibiotics tested, such as Levofloxacin, Cefuroxime, Ciprofloxacin, Tobramycin, and Amoxicillin/clavulanic acid. Among the *Klebsiella* isolates, those with the highest resistance were UJA K72 (resistant to 17 antibiotics) and UJA K1, UJA K2, UJA K3, UJA K5, UJA K14, and UJA K15, showing resistance to 15 of the antibiotics tested. On the other hand, isolates UJA K12 and UJA K13 were the least resistant, with just seven antibiotics in their resistance profile.

Among the *E. coli* samples, the most antibiotic-resistant isolate was UJA E12, showing resistance to 12 antibiotics, followed by isolates UJA E7, UJA E82, UJA E93, and UJA E96 (10 antibiotics). The least resistant isolate among this group was UJA E8 (five antibiotics).

### 2.3. Genetic Determinants Coding for Antibiotic Resistance

The study of genes related to antibiotic resistance showed positive results in almost 100% of the isolates for at least two genetic determinants of resistance (*bla*_OXA-23_ and *bla*_NDM-1_) among the *A. baumannii* samples, one resistance gene (*bla*_CTX-M_) among *E. coli*, and two genetic determinants (*bla*_CTX-M_ and *aac(6′)-Ib*) among *Klebsiella*. In contrast, *P. aeruginosa* showed just one high-frequency antibiotic resistance gene (*dfrA*), which was present in 68.4% of the isolates studied.

A high percentage of the isolates mainly showed genes that were involved in resistance to beta-lactam antibiotics (*bla*) and tetracyclines (*tet*). β-lactamase resistance family genes (*bla*_GES_, *bla*_PSE_, *bla*_IMP_, *bla*_VIM_, *bla*_TEM_, *bla*_VEB_, *bla*_PER_) were present in the majority of the isolates studied (75%). *bla*_GES_ was the most frequent β-lactamase gene detected in *A. baumannii* (61.3% of the isolates), in comparison with *bla*_PSE_ and *bla*_IMP_ genes, which were the least detected in this bacterial genus (3.2% and 6.5%, respectively). *bla*_PSE_ was also the most frequently detected β-lactamase gene in *E. coli* isolates (56%), while *bla*_VIM_ (43.2%) and *bla*_TEM_ (37.8%) were the most frequently found among the β-lactamase genes detected in *K. pneumoniae.*

On the other hand, very low percentages of β-lactamase genes (10.5% *bla*_VIM_, 5.3% *bla*_PSE_, and 0% *bla*_IMP_, *bla*_TEM_, *bla*_PER_, *bla*_VEB_, and *bla*_GES_) were detected among the *P. aeruginosa* isolates.

Genes involved in resistance to tetracyclines (*tet* A, B, C, D, E, or G) were also found in almost all of the isolates studied. *tet* (B) was the most frequently detected gene in *A. baumannii* (64.51% of the isolates) in comparison with other *tet* genes, at 22.58% *tet* (E) and 16.12% *tet* (A). Among the isolates identified as *E. coli*, 40% *tet* (A) and 28% *tet* (B) were detected, and for *K. pneumoniae,* the most frequently found gene was *tet* (A) (51.35%), compared to the other two *tet* genes detected in this species: 13.51% *tet* (E) and 2.7% *tet* (D). In the case of *P. aeruginosa,* low percentages of the genes were found (21.05% *tet* (B), 10.5% *tet* (A), and 5.3% *tet* (E)).

Among the *A. baumannii* isolates, UJA A68 expressed the highest number of AMR genes (11 positive results), and several isolates expressed 9 AMR genes (UJA A6, UJA A38, UJA A66, UJA A69, UJA A70, UJA A92, UJA A101, UJA A107, UJA A108, UJA A110, UJA A115, UJA A117, UJA A119, and UJA A120). On the contrary, isolates UJA A58 and UJA A35 showed positive results for just one and two AMR genes, respectively. The *Pseudomonas* isolate showing the highest number of AMR genes was UJA P106, with positive results for seven AMR genes, followed by isolates UJA P102 and UJA P29, with five and four AMR genes, respectively. Nine of the isolates, in contrast, showed positive results for just one or two AMR genes. 

Among the *Klebsiella* isolates, UJA K12 stands out, showing positive results for 10 AMR genes, as well as UJA K21, which is positive for 9 resistance genes. Eight AMR genes were also detected in isolates UJA K7, UJA K22, UJA K30, UJA K65, and UJA K80. On the other hand, isolate UJA K2 showed a positive result just for the *aac(6′)-Ib* gene and isolate UJA K11 just for the *bla*_TEM_, *dfrA 12* and *oqxA* genes.

The *E. coli* isolate showing the highest number of AMR genes was UJA E7, with 11 positive results, as well as resistance to 10 antibiotics, as previously shown. Isolates UJA E13 and UJA E24 also stand out with nine AMR genes. In contrast, isolate UJA E94 showed only the *bla*_CTX-M_ and *mdfA* genes, and isolates UJA E8, UJA E71, and UJA E83 were positive for just four AMR genes.

When studying possible associations between phenotypic resistances and the presence of genetic resistance determinants, a high percentage of the analyzed *A. baumanii* isolates were found to be positive for ampicillin resistance and also carried antibiotic resistance genes, especially those involved in resistance to beta-lactam antibiotics. Statistically significant associations were detected between ampicillin resistance and the presence of the *bla*_VEB_ gene (*p* = 0.023) and *bla*_GES_ gene (*p* = 0.029). Similar results were obtained for cefotaxime resistance and the presence of the *bla*_VEB_ gene (*p* = 0.023) and *bla*_GES_ gene (*p* = 0.029). A significant association was also found between the aztreonam resistance phenotype and the presence of the *bla*_GES_ gene in *E. coli* samples (*p* = 0.01). 

## 3. Discussion

The presence and heterogeneity of antimicrobial resistance genes described in clinically significant bacterial isolates and their potential role of reducing the efficacy of classically effective antibiotics represent a major challenge in the molecular detection of antimicrobial resistance functions and hinder the selection of appropriate antibiotics to treat drug-resistant infections.

Carbapenems, broad-spectrum antimicrobials that are highly stable against most β-lactamases, play a crucial role in the treatment of nosocomial infections caused by Gram-negative bacteria [28] and are often the antimicrobials of choice in the treatment of *A. baumannii* infections. However, their use has led to the development of resistance [29], mainly through the production of β-lactamases, alterations in the outer membrane protein, production of penicillin-binding proteins, and an increased activity of efflux pumps [30]. The most prevalent mechanism of extended-spectrum cephalosporin and carbapenem resistance in *A. baumannii* is enzymatic degradation by β-lactamases, mainly mediated through acquired carbapenem-hydrolyzing class D β-lactamases (oxacillinases) [31,32], as our results of 100% of the positive samples of this specie harboring *bla_OXA-23_* have corroborated. However, class B metallo-β-lactamases (MBLs) have also been reported in CRAB isolates [33]. 

Another resistance mechanism is based on the presence of clavulanic acid-inhibited extended-spectrum β-lactamases (ESBLs) that comprise PER-, VEB-, MBL-, VIM-, and IMP-type genes [34,35], as well as the Ambler class A carbapenemase GES, which is also described in *A. baumannii* [36,37,38]. Our research describes significant associations between ampicillin and cefotaxime resistance in *A. baumannii* and the presence of the *bla*_VEB_ and *bla*_GES_ genes. GES-type β-lactamase was originally identified as a cephamycin-hydrolyzing extended-spectrum β-lactamase family, and nowadays, twenty-six variants of the GES group have been identified, some of which are classified as carbapenemases and considered to be mainly responsible for carbapenem resistance in *A. baumannii* [39].

With respect to the significant association found in the present study between the aztreonam resistance phenotype and the presence of the *bla*_GES_ gene in *E. coli*, this also supports the recent worldwide report of *bla*_GES_ as a carbapenemase harbored by *K. pneumoniae, E. coli*, and *P. aeruginosa* [39,40,41]. Mehrotra et al. (2023) [12] also previously described *bla*_CTX-M-15_, *bla*_CMY-42_, *bla*_NDM-5_, and *aadA(2)* as prevalent in *E. coli*, and we have also detected a high presence of *bla*_PSE_ in these isolates. They also described *bla*_TEM-1B_, *bla*_OXA-232_, *bla*_NDM-1_, *rmtB,* and *rmtC* as predominant in *K. pneumoniae*, while we also detected *bla*_VIM_ and *bla*_TEM_ as frequently found in this species. In contrast, *P. aeruginosa* and *A. baumannii* have been described to predominantly harbor *bla*_VEB_, *bla*_VIM-2_, or *bla*_OXA-23_, among others [12].

Resistome-wide association studies have previously scored 46 markers for resistance to levofloxacin, amikacin, and meropenem in a panel of 672 *P. aeruginosa* strains, including representatives of globally disseminated MDR and XDR clones [42]. The *Bla*_OXA-48_ and *bla*_NDM-1_ genes correlate with carbapenem resistance in *Enterobacteriaceae* [43], and a high prevalence of OXA-type genes also induces the occurrence of MDR and XDR strains among clinical isolates of *A. baumannii* and seems to play a key role in biofilm formation by these bacteria [44].

When searching for genes that are responsible for tetracycline resistance, our results in *A. baumannii* agree with previous studies on clinical isolates from Spain [45], which revealed that the *tet* (B) gene, which affects both tetracycline and minocycline resistances, has a higher prevalence than the *tet* (A) gene, which affects only tetracycline, although it is known that the resistance to tetracycline in clinical isolates of *A. baumannii* is greater than that to minocycline. In fact, only two of the species analyzed in our study were found to be resistant to minocycline (UJA A1 and UJA A27). The *tet* (B) gene was also previously detected in high percentages of resistant isolates of *A. baumannii* in Iran [46,47] or, more recently, in Taiwan [48]. 

On the other hand, *P. aeruginosa* was the species with the lowest detected *bla* and *tet* genes, although high levels of resistance were described in most of the isolates studied. It has been previously described that a significant number of isolates of these bacteria are resistant to β-lactams, hindering the treatment of infections and leading to worse outcomes for patients. Moreover, the resistance in *P. aeruginosa* has proven to be more complex, as it might involve multiple known and possibly unknown resistance mechanisms [49]. In *P. aeruginosa*, resistance to carbapenems can be achieved either by the production of acquired carbapenemases or by hyperproduction of the cephalosporinase AmpC. In addition, nonenzymatic mechanisms such as the modification or inactivation of the porin OprD, increased *mexXYoprM* expresion, or the upregulation of different chromosomally encoded efflux pumps, have also been described as common mechanisms that are responsible for these resistances [50]. Mutations in genes that regulate biofilm production are also observed in many clinical isolates of *P. aeruginosa* and may reduce the susceptibility of this bacteria to β-lactams [51,52], so many mechanisms seem to be involved in *P. aeruginosa*’s resistance to antimicrobials, in addition to *bla* or *tet* genetic determinants.

With regard to *K. pneumoniae*, our results agree with the report on the two extensively drug-resistant *K. pneumoniae* strains collected in Italy and harboring *bla_VIM-1_* and *tet* (A) determinants, among others [53], as well as the presence of *bla*_TEM_ genes associated with high levels of resistance in this species that was described in a Portuguese hospital by Carvalho et al. (2021) [54]. These results support the change in the global epidemiological situation regarding this major pathogen, wcich is implicated in nosocomial infections [55]. Moreover, to date, 43 different *bla*_NDM_ gene variants have been reported, and the emergence of a novel *bla*_NDM-23_ allele from a *bla*_NDM-1_ ancestor that has disseminated it through a *Klebsiella pneumoniae* (ST437 clone) in several Spanish hospitals has recently been described [56]. In this study, 100% of our samples also showed positive results for the presence of the *aac(6′)-Ib* gene, which confirms the emergence of aminoglycoside resistance mediated by this factor among *Klebsiella* strains [57]. 

We also found several *tet* genes that are commonly found in clinical *E. coli* isolates, as previously described in urine samples from the Turkish population [58], or in clinical and even associated with nonclinical sources in Nigeria, which could be indicative of a potential reservoir of this resistance that may favor the worldwide distribution of tetracycline resistance and hence limit the reintroduction of this antibiotic even in combination therapy [59]. However, employing whole-genome sequencing should allow for the detection of additional resistance determinants, as well as mutations that are responsible for the observed phenotypes.

## 4. Materials and Methods

### 4.1. Clinical Samples and Bacterial Isolates

A collection of 112 clinical isolates were selected as multidrug-resistant Gram-negative bacteria, based on agar disk diffusion tests previously performed in the Microbiology Unit of the Academic Hospital in Jaén (Jaén, Spain). Multi-resistant bacteria were isolated from appropriate biological samples on agar plates with selective media: BD Pseudogel Agar (Becton Dickinson GmbH, Heidelberg, Germany) was used for the isolation of *Pseudomonas*, TBX Agar (Sigma-Aldrich, Saint Louis, MO, USA) was used to isolate *E. coli* samples, CHROMagar™ *Acinetobacter* was used for the recovery of *A. baumanni,* and CHROMagar™ ESBL was used to detect ESBL producers. Once isolated, bacteria were allowed to grow on blood agar plates for 24 h at 37 °C. These bacterial isolates were preserved with 20% glycerol at −80 °C for further assays.

### 4.2. Species Identification

#### 4.2.1. MALDI-TOF Spectrometry

A preliminary species identification was assessed by matrix-assisted laser desorption/ionization time of flight mass spectrometry (MALDI-TOF/Vitek-MS ref. 8290190, Biomérieux, Marcy-l’Étoile, France).

#### 4.2.2. r16S V3–V5 Sequence Identification and Analysis

Selected bacteria were analyzed by an amplified 16S ribosomal DNA V3-V4 hypervariable region. Total DNA from bacterial isolates was extracted using a commercial Kit for DNA extraction Genomic (Healthcare, Piscataway, NJ, USA) and a Puregene DNA isolation kit (QIAGEN Inc., Valencia, CA, USA) according to the manufacturer’s instructions. The 16S ribosomal DNA V3-V4 region was studied by PCR assays using one primer pair (V3fwd: 5′ AGAGTTT-GATCMTGGCTC 3′, V4rev: 5′ CNCGTCCTTCATCGCCT 3′) [60,61]. Thermal cycling consisted of initial denaturation at 94 °C for 4 min, followed by 30 cycles of denaturation at 96 °C for 10 s, annealing at 52 °C for 30 s, and extension at 72 °C for 2 min. A final extension step was carried out at 72 °C for 4 min. Ten microliters of reaction mix containing a PCR product was analyzed by electrophoresis in 1% (*w/v*) agarose (Sigma). PCR products were purified using QIAamp© DNA Stool Kit (QIAGEN), and sequencing was performed at Lifesequencing (Valencia, Spain). All sequences obtained were identified. A search for the homology of the DNA sequence was performed using the Basic Local Alignment Search Tool (BLAST) (https://blast.ncbi.nlm.nih.gov/Blast.cgi, accessed on 4 April 2024) at the National Center for Biotechnology Information, Bethesda, MD, USA.

### 4.3. PCR Screening for Resistance Genes

DNA from each of the analyzed isolates was used as a template in PCR assays using 24 primer pairs for resistance genes: 11 primer pairs were based on the nucleotide sequences of Beta-lactamases genes (*bla*), 1 primer pair was used for Aminoglycoside resistance gene (*aac*) identification, 6 primer pairs were used for Tetracycline resistance (*tet*), 3 primer pairs were used for Sulfonamide and Trimethoprim resistance (*sul*, *dfr*), and 2 primer pairs were used for Export pumps (*mdf*, *oxq*) (Table 6).

The PCR reaction mixtures (50 µL) contained 2.5 µL of each primer, 6 µL dNTPs, 5 µL Taq buffer, 0.2 U Taq polymerase, 3 µL 25 mM MgCl_2_, 28.8 µL H_2_O, and 2 µL of DNA. Ten microliters of reaction mix containing a PCR product was analyzed by electrophoresis in 1.5% (*w/v*) agarose (Sigma). The DNA amplification programs for each gene group were as follows:

Beta-lactamases genes (*bla*_TEM_, *bla*_PSE_, *bla*_CTX-M_, *bla*_CTX-M2_, *bla*_VIM_, *bla*_IMP_, *bla*_NDM_): for the PCR reaction, the PCR mixture was incubated for five minutes at 94 °C as an initial denaturation step, followed by 35 cycles of successive alternating temperatures as follows: denaturation step at 94 °C for 30 s, annealing step at 52 °C for 1 min, and extension step at 72 °C for 1 min. A final extension step at 72 °C for eight minutes was allowed.

Class D beta-lactamases (*bla*_OXA_): the PCR mixture was incubated for an initial denaturation step (95 °C, 5 min), followed by 35 cycles of denaturation (95 °C, 45 s), annealing (58 °C, 30 s), and extension (72 °C, 1 min), and a single final extension of 8 min at 72 °C. 

Broad-spectrum beta-lactamases (*bla*_PER_, *bla*_VEB_, *bla*_GES_): these were treated for an initial denaturation step (94 °C, 5 min), followed by 36 cycles of denaturation (94 °C, 1 min), annealing (55 °C, 1 min), and extension (72 °C, 45 s), and a single final extension step at 72 °C for 5 min was allowed.

Aminoglycoside resistance (*aac(6′)-Ib*): this was assessed through an initial denaturation step (94 °C, 3 min), followed by 43 cycles of denaturation (94 °C, 45 s), annealing (55 °C, 45 s), and extension (72 °C, 45 s), and a single final extension of 8 min at 72 °C.

Tetracycline resistance (*tet* (A), *tet* (B), *tet* (C), *tet* (D), *tet* (E), *tet* (G)): this was assessed through an initial denaturation step (94 °C, 3 min), followed by 35 cycles of denaturation (94 °C, 1 min), annealing (55 °C, 1 min), and extension (72 °C, 1 min), and a single final extension of 1 min at 72 °C.

Sulfonamide and trimethoprim resistance (*sul1, dfrA12, dfrA15*): these were assessed through an initial denaturation step (94 °C, 3 min), followed by 35 cycles of denaturation (94 °C, 40 s), annealing (55 °C, 30 s for genes *dfrA12* and *dfrA15*/65 °C, 30 s for gene *sul1*), and extension (72 °C, 1 min), and a single final extension of 8 min at 72 °C.

Efflux pumps (*mdfA, oxqA*): the PCR mixture for *mdfA* was incubated for an initial denaturation step (94 °C, 2 min), followed by 35 cycles of denaturation (94 °C, 1 min), annealing (58 °C, 1 min), and extension (72 °C, 30 s), and a single final extension of 2 min at 72 °C.

The PCR mixture for *oxqA* was incubated for three minutes at 94 °C as an initial denaturation step, followed by 35 cycles of successively alternating temperatures as follows: denaturation step at 94 °C for thirty seconds, annealing step at 58 °C for forty-five seconds, and extension step at 72 °C for one minute. A final extension step at 72 °C for eight minutes was allowed.

### 4.4. Statistics

The odds ratio (OR) and exact 95% confidence intervals were determined in order to evaluate the association between genetic determinants for antibiotic resistance and the phenotypic antibiotic resistance observed in antimicrobial resistance tests. An OR < 1 indicated a negative association and OR > 1 a positive association. The significance of the association between genetic determinants and phenotypic resistance to antibiotics was analyzed by Fisher’s test (*p* < 0.05).

## 5. Conclusions

The persistent evolution of resistance determinants and their successful spread among bacteria, with the consequent loss of antibiotic effectiveness, is challenging both for clinical practice and public health and therefore requires global actions. Data on the evolution and mechanisms of resistance in these pathogens may help limit the spread of these infections. In this study, 112 multidrug-resistant isolates from clinical samples were analyzed both phenotypically and genotypically to search for high resistance profiles among four genera. Most of the analyzed *Acinetobacter* and *Pseudomonas* isolates in this study did not show sensitivity to any of the antibiotics tested. Moreover, in the *Escherichia* and *Klebsiella* isolates, sensitivity to only a few of the antibiotics tested, such as Imipenem, Meropenem, Tigecycline, Cefoxitin, and Colistin, was observed. These multidrug-resistant isolates are causing an increase in mortality rates and creating a major challenge for physicians and healthcare workers regarding the eradication of either hospital- or community-based infections. Our research reveals the associations between ampicillin and cefotaxime resistance in *A. baumannii* and the presence of the *bla*_VEB_ and *bla*_GES_ genes, as well as between the aztreonam resistance phenotype and the presence of the *bla*_GES_ gene in *E. coli*. We also describe new prevalent genetic determinants in the four Gram-negative genera studied. These results may be useful for achieving better control in infection strategies and antibiotic management, particularly in some clinical scenarios, such as nosocomial infections in intensive care units.

## Figures and Tables

**Table 1:** Origin of clinical samples studied.

Species	Types of Samples						
	Respiratory Sample	Urine	Blood	Body Swab	Body Fluids	Exudates	Catheter
*Acinetobacter baumannii*	38.8%	19.3%	9.6%	6.5%	13%	6.4%	6.4%
*Escherichia coli*	8%	72%	12%	-	4%	4%	-
*Klebsiella pneumoniae*	8.7%	39.1%	26.2%	-	8.7%	13%	4.3%
*Pseudomonas aeruginosa*	52.6%	15.8%	5.3%	-	21%	5.3%	-

Respiratory samples (bronchial aspirate, tracheal aspirate, sputum, and respiratory samples), urine (urine, mid-micturition urine, bladder puncture urine, bladder catheter urine, and ureter urine), blood (blood, arterial blood, central venous catheter blood, venipuncture blood, and blood culture), body swab (axillary swab, pharyngeal swab, rectal swab, and rectal examination), body fluids (ascitic fluid, cerebrospinal fluid, peritoneal fluid, and pleural fluid), exudates (eschar exudate, wound exudate, and rectal exudate), and catheter (catheter, drainage, and abdominal drainage).

**Table 2:** Phenotypic and genotypic profiles of *Acinetobacter baumannii* isolates.

Isolate	Antimicrobial Resistance	Genetic Determinants
UJA A1	AMP, TIC, TZP, CAZ, CTX, FEP, ATM, IMP, MEM, DOR, GM, TOBRA, AMK, CIP, LEV, MINO, SXT	*bla*_OXA-23_, *bla*_NDM-1_, *tet* (B), *tet* (A), *tet* (E), *aac(6′)-Ib*, *bla*_VEB_
UJA A2	AMP, TIC, TZP, CAZ, CTX, FEP, ATM, IMP, MEM, DOR, GM, AMK, CIP, LEV, SXT	*bla*_OXA-23_, *bla*_NDM-1_, *tet* (B), *tet* (A), *tet* (E), *aac(6’)-Ib*, *bla*_VEB_, *bla_GES_*
UJA A6	AMP, TIC, TZP, CAZ, CTX, FEP, ATM, IMP, MEM, DOR, GM, AMK, CIP, LEV, SXT, CS	*bla*_OXA-23_, *bla*_NDM-1_, *tet* (B), *tet* (A), *tet* (E), *dfrA 12*, *aac(6′)-Ib*, *bla*_VEB_, *bla*_GES_
UJA A25	AMP, TIC, TZP, CAZ, CTX, FEP, ATM, IMP, MEM, DOR, GM, AMK, CIP, LEV, SXT	*bla*_OXA-23_, *bla*_NDM-1_, *tet* (E), *dfrA 12*, *aac(6′)-Ib*, *bla*_VEB_, *bla*_GES_
UJA A27	AMP, TIC, TZP, CAZ, CTX, FEP, ATM, DOR, GM, AMK, CIP, LEV, MINO, SXT	*bla*_CTX-M_, *bla*_OXA-23_, *bla*_NDM-1_, *tet* (B), *tet* (E), *aac(6′)-Ib*, *bla*_VEB_, *bla*_GES_
UJA A35	TZP, CAZ, FEP, ATM, IMP, MEM, GM, AMK, CIP, LEV, CS	*bla*_CTX-M_, *aac(6′)-Ib*
UJA A38	AMP, TIC, TZP, CAZ, CTX, FEP, ATM, IMP, MEM, DOR, GM, TOBRA, AMK, CIP, LEV, SXT, CS	*bla*_CTX-M_, *bla*_IMP_, *bla*_OXA-23_, *bla*_NDM-1_, *tet* (B), *dfrA 12*, *aac(6′)-Ib*, *bla*_VEB_, *bla*_GES_
UJA A39	AMP, TIC, TZP, CAZ, CTX, FEP, ATM, IMP, MEM, DOR, GM, AMK, CIP, LEV, SXT, CS	*bla*_CTX-M_, *bla*_OXA-23_, *bla*_NDM-1_, *tet* (B), *aac(6′)-Ib*, *bla*_VEB_, *bla*_GES_
UJA A40	AMP, TIC, TZP, CAZ, CTX, FEP, ATM, IMP, MEM, DOR, GM, AMK, CIP, LEV, SXT	*bla*_OXA-23_, *bla*_NDM-1_, *tet* (B), *aac(6′)-Ib*, *bla*_VEB_, *bla*_GES_
UJA A41	AMP, TIC, TZP, CAZ, CTX, FEP, ATM, IMP, MEM, DOR, GM, AMK, CIP, LEV, SXT	*bla*_CTX-M_, *bla*_OXA-23_, *bla*_NDM-1_, *tet* (B), *bla*_VEB_, *bla*_GES_
UJA A42	AMP, TIC, TZP, CAZ, CTX, FEP, ATM, IMP, MEM, DOR, GM, AMK, CIP, LEV, SXT	*bla*_CTX-M_, *bla*_OXA-23_, *bla*_NDM-1_, *tet* (B), *aac(6′)-Ib*, *bla*_VEB_, *bla*_GES_
UJA A50	AMP, TIC, TZP, CAZ, CTX, FEP, ATM, IMP, MEM, DOR, GM, AMK, CIP, LEV, SXT	*bla*_CTX-M_, *bla*_OXA-23_, *bla*_NDM-1_, *tet* (B), *dfrA 12*, *aac(6′)-Ib*, *bla*_VEB_, *bla*_GES_
UJA A51	AMP, TIC, TZP, CAZ, CTX, FEP, ATM, IMP, MEM, DOR, GM, AMK, CIP, LEV, SXT	*bla*_CTX-M_, *bla*_IMP_, *bla*_OXA-23_, *bla*_NDM-1_, *dfrA 12*, *bla*_VEB_, *bla*_GES_
UJA A52	AMP, TIC, TZP, CAZ, CTX, FEP, ATM, IMP, MEM, DOR, GM, AMK, CIP, LEV, SXT	*bla*_OXA-23_, *bla*_NDM-1_, *tet* (B), *dfrA 12*, *bla*_VEB_, *bla*_GES_
UJA A53	AMP, TIC, TZP, CAZ, CTX, FEP, ATM, MEM, DOR, GM, AMK, CIP, LEV, SXT	*bla*_CTX-M_, *bla*_OXA-23_, *bla*_NDM-1_, *dfrA 12*, *bla*_VEB_, *bla*_GES_
UJA A58	AMP, TIC, TZP, CAZ, CTX, FEP, ATM, IMP, MEM, DOR, GM, AMK, CIP, LEV, SXT, CS	*dfrA 12*
UJA A62	TZP, CAZ, FEP, ATM, GM, TOBRA, AMK, CIP, LEV	*bla*_OXA-23_, *bla*_NDM-1_, *tet* (B), *dfrA 12*,
UJA A64	TZP, CAZ, FEP, ATM, IMP, MEM, GM, AMK, CIP, LEV	*bla*_CTX-M_, *bla*_OXA-23_, *bla*_NDM-1_, *tet* (B), *aac(6′)-Ib*, *bla*_VEB_, *bla*_GES_
UJA A66	TZP, CAZ, FEP, ATM, IMP, MEM, GM, AMK, CIP, LEV	*bla*_CTX-M_, *bla*_OXA-23_, *bla*_NDM-1_, *tet* (B), *dfrA 12*, *aac(6′)-Ib*, *oqxA*, *bla*_VEB_, *bla*_GES_
UJA A68	TZP, CAZ, FEP, ATM, IMP, MEM, GM, AMK, CIP, LEV	*bla*_CTX-M_, *bla*_OXA-23_, *bla*_NDM-1_, *tet* (B), *tet* (A), *dfrA 12*, *aac(6′)-Ib*, *mdfA*, *oqxA*, *bla*_VEB_, *bla*_GES_
UJA A69	TZP, CAZ, FEP, ATM, IMP, MEM, GM, TOBRA, AMK, CIP, LEV	*bla*_CTX-M_, *bla*_OXA-23_, *bla*_NDM-1_, *dfrA 15*, *dfrA 12*, *sul1*, *aac(6′)-Ib*, *bla*_VEB_, *bla*_GES_
UJA A70	TZP, CAZ, FEP, ATM, IMP, MEM, GM, AMK, CIP, LEV	*bla*_CTX-M_, *bla*_OXA-23_, *bla*_NDM-1_, *dfrA 15*, *dfrA 12*, *sul1*, *aac(6′)-Ib*, *bla*_VEB_, *bla*_GES_
UJA A92	TZP, CAZ, FEP, ATM, IMP, MEM, GM, AMK, CIP, LEV	*bla*_CTX-M_, *bla*_OXA-23_, *bla*_NDM-1_, *dfrA 15*, *dfrA 12*, *sul1*, *aac(6′)-Ib*, *bla*_VEB_, *bla*_GES_
UJA A101	TZP, CAZ, FEP, ATM, GM, TOBRA, CIP, LEV	*bla*_CTX-M_, *bla*_OXA-23_, *bla*_NDM-1_, *dfrA 15*, *dfrA 12*, *sul1*, *aac(6′)-Ib*, *bla*_VEB_, *bla*_GES_
UJA A107	TZP, CAZ, FEP, ATM, IMP, MEM, GM, AMK, CIP, LEV	*bla*_CTX-M_, *bla*_OXA-23_, *bla*_NDM-1_, *dfrA 15*, *dfrA 12*, *sul1*, *aac(6′)-Ib*, *bla*_VEB_, *bla*_GES_
UJA A108	TZP, CAZ, FEP, ATM, IMP, MEM, GM, AMK, CIP, LEV	*bla*_CTX-M_, *bla*_OXA-23_, *bla*_NDM-1_, *dfrA 15*, *dfrA 12*, *sul1*, *aac(6′)-Ib*, *bla*_VEB_, *bla*_GES_
UJA A110	TZP, CAZ, FEP, ATM, MEM, GM, AMK, CIP, LEV	*bla*_CTX-M_, *bla*_OXA-23_, *bla*_NDM-1_, *dfrA 15*, *dfrA 12*, *sul1*, *aac(6′)-Ib*, *bla*_VEB_, *bla*_GES_
UJA A115	AMP, TIC, TZP, CAZ, CTX, FEP, ATM, IMP, MEM, DOR, AMK, CIP, LEV, SXT	*bla*_CTX-M_, *bla*_OXA-23_, *bla*_NDM-1_, *dfrA 15*, *dfrA 12*, *sul1*, *aac(6′)-Ib*, *bla*_VEB_, *bla*_GES_
UJA A117	TZP, CAZ, FEP, ATM, IMP, MEM, GM, AMK, CIP, LEV	*bla*_CTX-M_, *bla*_OXA-23_, *bla*_NDM-1_, *dfrA 15*, *dfrA 12*, *sul1*, *aac(6′)-Ib*, *bla*_VEB_, *bla*_GES_
UJA A119	TZP, CAZ, FEP, ATM, GM, TOBRA, AMK, CIP, LEV	*bla*_CTX-M_, *bla*_OXA-23_, *bla*_NDM-1_, *dfrA 15*, *dfrA 12*, *sul1*, *aac(6′)-Ib*, *bla*_VEB_, *bla*_GES_
UJA A120	TZP, CAZ, FEP, ATM, IMP, MEM, GM, AMK, CIP, LEV	*bla*_CTX-M_, *bla*_OXA-23_, *bla*_NDM-1_, *dfrA 15*, *dfrA 12*, *sul1*, *aac(6′)-Ib*, *bla*_VEB_, *bla*_GES_

Nalixylic Acid: NAL, Amikacin: AMK, Amoxicillin/Clavulanic Acid: AMC, Ampicillin: AMP, Ampicillin-Sulbactam: SAM, Aztreonam: ATM, Cephalothin: CFL, Cefepime: FEP, Cefotaxime: CTX, Cefoxitin: FOX, Ceftacidime: CAZ, Ceftolozane-Tazobactam: TZC, Cefuroxime: CXM, Ciprofloxacin: CIP, Chloramphenicol: CHL, Colistin: CS, Doripenem: DOR, Ertapenem: ETP, Fosfomycin: FOS, Gentamicin: GM, Imipenem: IMP, Levofloxacin: LEV, Meropenem: MEM, Minocycline: MINO, Nitrofurantoin: NIT, Norfloxacin: NOR, Piperacillin: PIP, Piperacillin/Tazobactam: TZP, Ticarcillin: TIC, Tigecycline: TGC, Tobramycin: TOBRA, Trimethoprim-Sulfamethoxazole: SXT.

**Table 3:** Phenotypic and genotypic profiles of *Escherichia coli* isolates.

Isolate	Antimicrobial Resistance	Genetic Determinants
UJA E4	AMP, CXM, CIP, LEV, SXT, NAL, NOR	*bla*_CTX-M_, *bla*_IMP_, *bla*_TEM_, *tet* (A), *sul1*, *mdfA*
UJA E7	AMP, AMC, CXM, GM, TOBRA, AMK, CIP, LEV, NOR, SXT	*bla*_CTX-M_, *bla*_IMP_, *bla*_Vim-2_, *bla*_OXA-23_, *bla*_NDM-1_, *bla*_PSE_, *bla*_TEM_, *tet (B)*, *dfrA 12*, *aac(6′)-Ib*, *mdfA*
UJA E8	AMP, CXM, CTX, CIP, SXT	*bla*_CTX-M_, *bla*_Vim-2_, *bla*_TEM_, *mdfA*
UJA E9	AMP, AMC CXM, CIP, LEV, SXT, CHL	*bla*_CTX-M_, *bla*_OXA-23_, *bla*_NDM-1_, *bla*_PSE_, *tet (B)*, *tet* (A), *sul1*, *mdfA*
UJA E12	AMP, AMC, CXM, FEP, GM, TOBRA, CIP, LEV, SXT, FT, CHL, FOS	*bla*_CTX-M_, *bla*_IMP_, *bla*_PSE_, *tet* (A), *dfrA 12*, *sul1*, *aac(6′)-Ib*, *mdfA*
UJA E13	AMP, AMC, CXM, CTX, FEP, TOBRA, CIP, SXT	*bla*_CTX-M_, *bla*_Vim-2_, *bla_NDM-1_, bla*_PSE_, *bla*_TEM_, *tet A*, *sul1*, *aac(6′)-Ib*, *mdfA*
UJA E15	AMP, AMC, TZP, CXM, CAZ, CTX, FEP	*bla*_CTX-M_, *bla*_Vim-2_, *bla*_PSE_, *bla*_TEM_, *tet (B)*, *sul1*, *aac(6′)-Ib*, *mdfA*
UJA E17	AMP, TZP, CXM, FEP, GM, TOBRA, CIP, LEV	*bla*_CTX-M_, *bla*_Vim-2_, *bla*_NDM-1_, *bla*_PSE_, *bla*_TEM_, *sul1*, *aac(6′)-Ib*, *mdfA*
UJA E19	AMP, AMC, CXM, GM, TOBRA, NOR, CIP, LEV, SXT	*bla*_CTX-M_, *bla*_IMP_, *bla*_Vim-2_, *bla*_NDM-1_, *bla*_PSE_, *bla*_TEM_, *tet B*, *tet A*, *dfrA 12*, *sul1*, *aac(6′)-Ib*, *mdfA*
UJA E24	AMP, AMC, CXM, CTX, GM, CIP, SXT, FOS	*bla*_CTX-M_, *bla*_IMP_, *bla*_NDM-1_, *bla*_PSE_, *bla*_TEM_, *dfrA 12*, *sul1*, *aac(6′)-Ib*, *mdfA*
UJA E26	AMP, AMC, CXM, TOBRA, NOR, CIP, LEV, SXT, FOS	*bla*_CTX-M_, *bla*_PSE_, *dfrA 12*, *sul1*, *aac(6′)-Ib*, *mdfA*
UJA E63	AMP, AMC, TZP, CXM, CAZ, CTX, FEP	*bla*_CTX-M_, *bla*_IMP_, *dfrA 12*, *aac(6′)-Ib*, *mdfA*
UJA E71	AMP, AMC, TZP, CXM, FEP, TOBRA, CIP, LEV	*bla*_CTX-M_, *bla_IMP_*, *dfrA 12*, *bla_VEB_*
UJA E79	AMP, CXM, CTX, FEP, TOBRA, CIP, FOS, ATM	*bla*_CTX-M_, *bla*_IMP_, *dfrA 15*, *dfrA 12*, *mdfA*, *bla_GES_*
UJA E82	AMP, AMC, TZP, CXM, GM, TOBRA, NOR, CIP, LEV, SXT	*bla*_CTX-M_, *bla*_IMP_, *bla*_Vim-2_, *bla*_PSE_, *dfrA 12*, *sul1*, *aac(6′)-Ib*, *mdfA*
UJA E83	AMP, AMC, CXM, TOBRA, NIT, NOR, CIP, LEV, SXT	*bla*_CTX-M_, *bla*_Vim-2_, *tet (B)*, *dfrA 12*
UJA E84	AMP, CXM, CTX, FEP, ATM, CIP, SXT	*bla*_CTX-M_, *bla*_IMP_, *bla*_PSE_, *tet* (B), *dfrA 12*, *aac(6′)-Ib*, *mdfA*, *bla_GES_*
UJA E87	AMP, AMC, CXM, CTX, FEP, CIP, ATM	*bla*_CTX-M_, *bla*_PSE_, *tet* (A), *dfrA 15*, *dfrA 12*, *sul1*, *mdfA*
UJA E88	AMP, AMC, CXM, CTX, FEP, GM, TOBRA, CIP	*bla*_CTX-M_, *bla*_Vim-2_, *dfrA 12*, *aac(6′)-Ib*, *mdfA*
UJA E89	AMP, CXM, GM, TOBRA, NOR, CIP, LEV, FOS	*bla*_CTX-M_, *bla*_Vim-2_, *tet* (A), *dfrA 12*, *aac(6′)-Ib*, *mdfA*
UJA E90	AMP, AMC, CXM, CTX, GM, CIP, SXT, FOS	*bla*_CTX-M_, *tet* (B), *tet* (A), *dfrA 12*, *mdfA*
UJA E91	AMP, AMC, CXM, CTX, GM, CIP, SXT, FOS	*bla*_CTX-M_, *dfrA 12*
UJA E93	AMP, AMC, TZP, GM, TOBRA, CIP, LEV, *CXM*, ETP, SXT	*bla*_CTX-M_, *bla*_PSE_, *tet* (A), *dfrA 15*, *dfrA 12*, *sul1*, *aac(6′)-Ib*
UJA E94	AMP, CXM, FEP, GM, TOBRA, CIP, LEV	*bla*_CTX-M_, *mdfA*
UJA E96	AMP, AMC, CXM, FEP, GM, TOBRA, AMK, CIP, LEV SXT	*bla*_CTX-M_, *bla*_PSE_, *tet* (A), *dfrA 12*, *sul1*, *aac(6′)-Ib*, *mdfA*

Nalixylic Acid: NAL, Amikacin: AMK, Amoxicillin/Clavulanic Acid: AMC, Ampicillin: AMP, Ampicillin-Sulbactam: SAM, Aztreonam: ATM, Cephalothin: CFL, Cefepime: FEP, Cefotaxime: CTX, Cefoxitin: FOX, Ceftacidime: CAZ, Ceftolozane-Tazobactam: TZC, Cefuroxime: CXM, Ciprofloxacin: CIP, Chloramphenicol: CHL, Colistin: CS, Doripenem: DOR, Ertapenem: ETP, Fosfomycin: FOS, Gentamicin: GM, Imipenem: IMP, Levofloxacin: LEV, Meropenem: MEM, Minocycline: MINO, Nitrofurantoin: NIT, Norfloxacin: NOR, Piperacillin: PIP, Piperacillin/Tazobactam: TZP, Ticarcillin: TIC, Tigecycline: TGC, Tobramycin: TOBRA, Trimethoprim-Sulfamethoxazole: SXT.

**Table 4:** Phenotypic and genotypic profiles of *Klebsiella pneumoniae* isolates.

Isolate	Antimicrobial Resistance	Genetic Determinants
UJA K1	AMP, AMC, TZP, CXM, CTX, FEP, ATM, IMP, GM, TOBRA, AMK, CIP, NIT, FOS, SXT	*bla*_CTX-M_, *bla*_TEM_, *tet* (A), *dfrA 12*, *aac(6′)-Ib*, *mdfA*, *oqxA*
UJA K2	AMP, AMC, TZP, CXM, CAZ, CTX, FEP, IMP, MEM, TOBRA, AMK, CIP, LEV, ETP, SXT	*aac(6′)-Ib*
UJA K3	AMP, AMC, TZP, CXM, CAZ, CTX, ATM, GM, TOBRA, AMK, CIP, LEV, ETP, FOS, SXT	*bla*_IMP_, *tet* (A), *dfrA 15*, *dfrA 12*, *aac(6′)-Ib*
UJA K5	AMP, AMC, TZP, CXM, CAZ, CTX, FEP, IMP, MEM, TOBRA, AMK, CIP, LEV, ETP, SXT	*bla*_TEM_, *tet* (A), *tet* (E), *dfrA 15*, *dfrA 12*, *aac(6′)-Ib*, *oqxA*
UJA K6	AMP, CXM, CAZ, CTX, GM, TOBRA, FOS, SXT	*bla*_CTX-M_, *tet* (A), *tet* (E), *dfrA 15*, *dfrA 12*, *oqxA*
UJA K7	AMP, AMC, TZP, CXM, CAZ, CTX, FEP, TOBRA, CIP, LEV, SXT	*bla*_CTX-M_, *bla*_PSE_, *bla*_TEM_, *tet A, tet* (E), *dfrA 12*, *aac(6′)-Ib*, *oqxA*
UJA K8	AMP, AMC, TZP, CXM, CAZ, CTX, GM, TOBRA, CIP, LEV, NIT, FOS, SXT	*bla*_CTX-M_, *bla*_TEM_, *tet* (A), *tet* (E), *dfrA 12*, *aac(6′)-Ib*, *oqxA*
UJA K9	AMP, AMC, TZP, CXM, CAZ, CTX, FEP, IMP, MEM, TOBRA, AMK, CIP, LEV, SXT	*bla*_TEM_, *dfrA 12*, *sul1*, *aac(6′)-Ib*, *oqxA*
UJA K10	AMP, AMC, TZP, CXM, CAZ, CTX, FEP, GM, TOBRA, CIP, LEV, TGC, ETP, SXT	*bla*_CTX-M_, *bla*_TEM_, *tet* (A), *aac(6′)-Ib*, *oqxA*
UJA K11	AMP, CXM, CAZ, CTX, FEP, GM, TOBRA, AMK, ETP	*bla*_TEM_, *dfrA 12*, *oqxA*
UJA K12	AMP, AMC, TZP, *CXM*, CIP, LEV, ETP	*bla*_CTX-M_, *bla*_TEM_, *tet* (A), *dfrA 12*, *aac(6′)-Ib*, *mdfA*, *oqxA*
UJA K13	AMP, AMC, TZP, FEP, CIP, LEV, SXT,	*bla*_TEM_, *dfrA 15*, *oqxA*
UJA K14	AMP, AMC, TZP, CXM, CAZ, CTX, FEP, IMP, MEM, TOBRA, AMK, CIP, LEV, ETP, SXT	*bla*_TEM_, *dfrA 15*, *dfrA 12*, *sul1*, *aac(6′)-Ib*, *oqxA*
UJA K15	AMP, AMC, TZP, CXM, CAZ, CTX, FEP, IMP, MEM, TOBRA, AMK, CIP, LEV, ETP, SXT	*dfrA 15*, *dfrA 12*, *sul1*, *aac(6′)-Ib*, *oqxA*, *bla*_VEB_
UJA K16	AMP, AMC, CXM, FEP, GM, TOBRA, CIP, LEV, SXT	*bla*_CTX-M_, *bla*_Vim-2_, *bla*_NDM-1_, *bla*_PSE_, *dfrA 12*, *sul1*, *aac(6′)-Ib*,
UJA K21	AMP, AMC, CXM, TZP, CAZ, FEP, CTX, IMP, MEM, GM, TOBRA, CIP, LEV, SXT	*bla*_CTX-M_, *bla*_Vim-2_, *bla*_PSE_, *bla*_TEM_, *tet* (A), *dfrA15*, *dfrA 12*, *sul1*, *aac(6′)-Ib*
UJA K22	AMP, AMC, TZP, CXM, FEP, GM, TOBRA, CIP	*bla*_CTX-M_, *bla*_Vim-2_, *bla_OXA-23_, bla*_NDM-1_, *bla*_PSE_, *dfrA 12*, *sul1*, *aac(6′)-Ib*
UJA K30	AMP, CXM, CTX, GM, TOBRA, NIT, CIP, SXT, FOS	*bla*_CTX-M_, *bla*_Vim-2_, *bla*_PSE_, *bla*_TEM_, *tet* (A), *dfrA15*, *sul1*, *aac(6′)-Ib*
UJA K32	AMP, AMC, CXM, GM, TOBRA, NOR, CIP, LEV, SXT	*bla*_CTX-M_, *bla*_Vim-2_, *tet* (A), *dfrA 12*, *aac(6′)-Ib*
UJA K33	AMP, CXM, CTX, GM, TOBRA, NIT, CIP, SXT, FOS	*bla*_CTX-M_, *bla*_Vim-2_, *bla*_PSE_, *bla*_TEM_, *sul1*, *aac(6′)-Ib*
UJA K34	AMP, AMC, TZP, CXM, FEP, GM, TOBRA, CIP, LEV, SXT	*bla*_CTX-M_, *bla*_Vim-2_, *bla*_OXA-23_, *bla*_NDM-1_, *bla*_TEM_, *tet A*, *aac(6′)-Ib*
UJA K44	AMP, AMC, TZP, CXM, FEP, GM, TOBRA, CIP, LEV, SXT	*bla*_CTX-M_, *tet* (A), *aac(6′)-Ib*, *oqxA*
UJA K55	AMP, AMC, CXM, FEP, GM, TOBRA, CIP, LEV, SXT	*bla*_CTX-M_, *bla*_Vim-2_, *dfrA 12*, *aac(6′)-Ib*, *oqxA*
UJA K59	AMP, AMC, TZP, CXM, IMP, ETP, TOBRA, NIT, NOR, CIP, LEV, SXT, FOS	*bla*_CTX-M_, *bla*_Vim-2_, *tet* (B), *dfrA 12*, *aac(6′)-Ib*, *oqxA*
UJA K61	AMP, AMC, TZP, CXM, FEP, GM, TOBRA, CIP, LEV	*bla*_CTX-M_, *bla*_Vim-2_, *dfrA 12*, *aac(6′)-Ib*, *oqxA*
UJA K65	AMP, AMC, TZP, CXM, FEP, GM, TOBRA, CIP, LEV, TGC, SXT	*bla*_CTX-M_, *bla*_Vim-2_, *tet* (B), *tet* (A), *dfrA 12*, *aac(6′)-Ib*, *oqxA*, *bla*_GES_
UJA K72	AMP, AMC, TZP, CXM, FOX, CAZ, CTX, ETP, GM, TOBRA, NIT, NOR, CIP, LEV, SXT, FOS, NAL	*bla*_CTX-M_, *bla*_Vim-2_, *tet* (A), *dfrA 12*, *aac(6′)-Ib*, *oqxA*
UJA K74	AMP, AMC, CXM, CAZ, CTX, FEP, GM, TOBRA, CIP, LEV, SXT	*bla*_CTX-M_, *bla*_Vim-2_, *dfrA 12*, *aac(6′)-Ib*, *oqxA*
UJA K76	AMP, AMC, TZP, CXM, CAZ, CTX, FEP, IMP, TOBRA, CIP, LEV, SXT	*bla*_CTX-M_, *bla*_Vim-2_, *tet* (D), *dfrA 15*, *dfrA 12*, *aac(6′)-Ib*
UJA K77	AMP, AMC, TZP, CXM, FOX, CAZ, CTX, FEP, ETP, GM, TOBRA, CIP, LEV, SXT	*bla*_CTX-M_, *bla*_Vim-2_, *bla_PSE_*, *dfrA 12*, *sul1*, *aac(6′)-Ib*
UJA K80	AMP, AMC, CXM, TOBRA, NOR, CIP, LEV, SXT, FOS	*bla*_CTX-M_, *bla*_Vim-2_, *bla*_PSE_, *tet* (A), *dfrA 15*, *dfrA 12*, *sul1*, *aac(6′)-Ib*
UJA K98	AMP, AMC, TZP, CXM, CAZ, CTX, FEP, IMP, MEM, GM, TOBRA, CIP, LEV, SXT	*bla*_CTX-M_, *bla*_PSE_, *dfrA 12*, *sul1*, *aac(6′)-Ib*, *mdfA*
UJA K109	AMP, AMC, TZP, CXM, FEP, GM, TOBRA, CIP, LEV	*bla*_CTX-M_, *tet* (A), *tet* (E), *dfrA 12*, *aac(6′)-Ib*
UJA K112	AMP, AMC, CXM, CTX, FEP, GM, TOBRA, NIT, CIP, SXT, ATM	*dfrA 15*, *dfrA 12*, *sul1*, *aac(6′)-Ib*
UJA K114	AMP, AMC, TZP, CXM, CAZ, CTX, FEP, GM, TOBRA, CIP, LEV, SXT	*bla*_CTX-M_, *bla*_PSE_, *tet* (A), *dfrA 15*, *dfrA 12*, *sul1*, *aac(6′)-Ib*
UJA K116	AMP, AMC, TZP, CXM, CAZ, CTX, FEP, IMP, MEM, GM, TOBRA, CIP, LEV, TGC, SXT	*bla*_CTX-M_, *bla*_PSE_, *dfrA 12*, *sul1*, *aac(6′)-Ib*
UJA K118	AMP, AMC, TZP, CXM, CAZ, CTX, FEP, IMP, MEM, GM, TOBRA, CIP, LEV, TGC, SXT	*bla*_CTX-M_, *bla*_PSE_, *tet* (A), *dfrA 12*, *aac(6′)-Ib*

Nalixylic Acid: NAL, Amikacin: AMK, Amoxicillin/Clavulanic Acid: AMC, Ampicillin: AMP, Ampicillin-Sulbactam: SAM, Aztreonam: ATM, Cephalothin: CFL, Cefepime: FEP, Cefotaxime: CTX, Cefoxitin: FOX, Ceftacidime: CAZ, Ceftolozane-Tazobactam: TZC, Cefuroxime: CXM, Ciprofloxacin: CIP, Chloramphenicol: CHL, Colistin: CS, Doripenem: DOR, Ertapenem: ETP, Fosfomycin: FOS, Gentamicin: GM, Imipenem: IMP, Levofloxacin: LEV, Meropenem: MEM, Minocycline: MINO, Nitrofurantoin: NIT, Norfloxacin: NOR, Piperacillin: PIP, Piperacillin/Tazobactam: TZP, Ticarcillin: TIC, Tigecycline: TGC, Tobramycin: TOBRA, Trimethoprim-Sulfamethoxazole: SXT.

**Table 5:** Phenotypic and genotypic profiles of *Pseudomonas aeruginosa* isolates.

Isolate	Antimicrobial Resistance	Genetic Determinants
UJA P3	IMP, MEM, CIP, LEV, FOS	*bla*_OXA-23_, *tet* (B)
UJA P28	TZP, CAZ, FEP, GM, CIP, LEV, TZC	*bla*_CTX-M_
UJA P29	AMP, TIC, PIP, TZP, CTX, FEP, IMP, MEM, CIP, LEV, TGC, MINO, SXT	*bla*_CTX-M_, *bla*_Vim-2_, *dfrA 12*, *aac(6′)-Ib*
UJA P36	SAM, AMC, CFL, CXM, FOX, CTX, FEP, IMP, MEM, ETP, GM, CIP, LEV, TGC, SXT	*bla*_Vim-2_
UJA P37	AMC, PIP, TZP, CAZ, CTX, FEP, IMP, MEM, DOR, GM, AMK, LEV, TGC, MINO, SXT	*dfrA 12*
UJA P45	TZP, IMP, MEM, GM, CIP, LEV	*bla*_CTX-M_, *dfrA 12*, *aac(6′)-Ib*
UJA P48	TZP, CAZ, FEP IMP, MEM	*dfrA 12*
UJA P54	SAM, PIP, TZP, CAZ, CTX, FEP, ATM, IMP, MEM, DOR, GM, AMK, CIP, LEV, TGC, MINO, SXT	*dfrA 12*
UJA P78	TZP, CAZ, FEP ATM, IMP, MEM, GM, AMK, CIP, LEV	*bla*_CTX-M_, *dfrA 12*, *aac(6′)-Ib*
UJA P95	TZP, CAZ, FEP, ATM, IMP, MEM, GM, CIP, LEV	*dfrA 12*
UJA P99	PIP, TZP, CAZ, CTX, FEP, ATM, IMP, MEM, GM, CIP, LEV	*tet* (B), *dfrA 12*, *aac(6′)-Ib*
UJA P100	TZP, CAZ, FEP, IMP, MEM, GM, AMK, CIP, LEV	*dfrA 12*, *aac(6′)-Ib*
UJA P102	TZP, CAZ, FEP, ATM, IMP, MEM, GM, CIP, LEV	*bla*_CTX-M_, *tet* (B), *dfrA 12*, *aac(6′)-Ib*, *mdfA*
UJA P103	TZP, CAZ, FEP, IMP, MEM, LEV	
UJA P104	TZP, CAZ, FEP, ATM, IMP, MEM, CIP, LEV	
UJA P105	TZP, CAZ, FEP, IMP, MEM, CIP, LEV	
UJA P106	TZP, CAZ, FEP, IMP, MEM, GM, CIP, LEV, TZC	*bla*_PSE_, *tet* (B), *tet* (E), *dfrA 12*, *sul1*, *aac(6′)-Ib*, *mdfA*
UJA P111	CAZ, FEP, ATM, TOBRA, CIP, LEV	*tet* (A), *dfrA 12, bla_PSE_*
UJA P113	TZP, CAZ, FEP, GM, CIP, LEV, TZC	*tet* (A), *dfrA 12*

Nalixylic Acid: NAL, Amikacin: AMK, Amoxicillin/Clavulanic Acid: AMC, Ampicillin: AMP, Ampicillin-Sulbactam: SAM, Aztreonam: ATM, Cephalothin: CFL, Cefepime: FEP, Cefotaxime: CTX, Cefoxitin: FOX, Ceftacidime: CAZ, Ceftolozane-Tazobactam: TZC, Cefuroxime: CXM, Ciprofloxacin: CIP, Chloramphenicol: CHL, Colistin: CS, Doripenem: DOR, Ertapenem: ETP, Fosfomycin: FOS, Gentamicin: GM, Imipenem: IMP, Levofloxacin: LEV, Meropenem: MEM, Minocycline: MINO, Nitrofurantoin: NIT, Norfloxacin: NOR, Piperacillin: PIP, Piperacillin/Tazobactam: TZP, Ticarcillin: TIC, Tigecycline: TGC, Tobramycin: TOBRA, Trimethoprim-Sulfamethoxazole: SXT.

**Table 6:** Primer pairs used for the screening of resistance genes.

Gene	Sequence	Product Size (bp)	References
*bla*_TEM_	5′-ATTCTTGAAGACGAAAGGGC-3′ 5′-ACGCTCAGTGGAACGAAAAG-3′	1150	[62]
*bla*_PSE_	5′-GGCAATCACACTCGATGATGCGT-3′ 5′-GGCTCAATCCGGTCTAGACGAGT-3′	156	[63]
*bla*_CTX-M_	5′-GGTTAAAAAATCACTGCGTC-3′ 5′-TTGGTGACGATTTTAGCCGC-3′	540	[64]
*bla*_CTX-M2_	5′-ATGATGACTCAGAGCATTCG-3′ 5′-TGGGTTACGATTTTCGCCGC-3′	859–876	[65]
*bla*_VIM_	5′-GTTTGGTCGCCATATCGCAAC-3′ 5′-ATTGCGCAGCACCAGGATAG-3′	801	[66]
*bla*_IMP_	5′-GAAGGCGTTTATGTTCATAC-3′ 5′-GTATGTTTCAAGAGTGATGC-3′	640	[66]
*bla*_NDM_	5′-GCAGCTTGTCGGCCATGCGGGC-3′ 5′-GGTCGCGAAGCTGAGCACCGCAT-3′	621	[67]
*bla*_OXA_	5′-AGCCGTTAAAATTAAGCCC-3′ 5′-CTTGATTGAAGGATTGGGCG-3′	438	[68]
*bla*_PER_	5′-AATTTGGGCTTAGGGCAGAA-3′ 5′-ATGAATGTCATTATAAAAGC-3′	933	[69]
*bla*_VEB_	5′-CGACTTCCATTTCCCGATGC-3′ 5′-GGACTCTGCAACAAATACGC-3′	642	[70]
*bla*_GES_	5′ -ATGCGCTTCATTCACGCAC-3′ 5′-CTATTTGTCCGTGCTCAGG-3′	860	[71]
*aac(6′)-Ib*	5′-AACAGCCTCAGCAGCCGGTTA-3′ 5′-TTCGCCGCAATCATCCCTAGC-3′	482	[72]
*tet* (A)	5′-GTAATTCTGAGCACTGTCGC-3′ 5′-CTGCCTGGACAACATTGCTT-3′	210	[73]
*tet* (B)	5′-CTCAGTATTCCAAGCCTTTG-3′ 5′-CTAAGCACTTGTCTCCTGTT-3′	659	[73]
*tet* (C)	5′-TCTAACAATGCGCTCATCGT-3′ 5′-GGTTGAAGGCTCTCAAGGGC-3′	418	[73]
*tet* (D)	5′-ATTACACTGCTGGACGCGAT-3′ 5′-CTGATCAGCAGACAGATTGC-3′	787	[73]
*tet* (E)	5′-GTGATGATGGCACTGGTCAT-3′ 5′-CTCTGCTGTACATCGCTCTT-3′	278	[73]
*tet* (G)	5′-GCTGCGCACCTGAAACTCCA-3′ 5′-AACCTCGTTCAACAGCTCTA-3′	468	[73]
*sul1*	5′-GGTGACGGTGTTCGGCATTC-3′ 5′-GCGAGGGTTTCCGAGAAGGTG-3′	436	[62]
*dfrA12*	5′-GGTGSGCAGAAGATTTTTCGC-3′ 5′-TGGGAAGAAGGCGTCACCCTC-3′	462	[62]
*dfrA15*	5′-GTGAAACTATCACTAATGG-3′ 5′-TTAACCCTTTTGCCAGATTT-3′	473	[62]
*mdfA*	5′-CATTGGCAGCGATCTCCTTT-3′ 5′-TTATAGTCACGACCGACTTCTTTCA-3′	103	[74]
*oxqA*	5′-CTCGGCGCGATGATGCT-3′ 5′-CCACTCTTCACGGGAGACGA-3′	670	[75]

## Data Availability

The original contributions presented in this study are included in the article; further inquiries can be directed to the corresponding author.
